# Endovascular Treatment for Fungal Internal Carotid Artery Aneurysm

**DOI:** 10.7759/cureus.89684

**Published:** 2025-08-09

**Authors:** Naoki Irizato, Tomohiko Ozaki, Hajime Nakamura, Masatoshi Takagaki, Hiroki Yamazaki, Toru Umehara, Haruhiko Kishima

**Affiliations:** 1 Department of Neurosurgery, The University of Osaka Graduate School of Medicine, Suita, JPN; 2 Department of Neurosurgery, Osaka International Cancer Institute, Osaka, JPN

**Keywords:** endovascular treatment, fungal internal carotid aneurysm, fungal sinusitis, invasive aspergillus, ruptured cerebral aneurysm

## Abstract

Fungal cerebral aneurysms, particularly those resulting from direct invasion by fungal sinusitis, are rare and often fatal when involving the cavernous segment of the internal carotid artery (ICA). We present a case of a ruptured fungal ICA aneurysm caused by *Aspergillus* sinusitis, successfully treated with parent artery occlusion (PAO).

In this case, an 80-year-old woman presented with right ptosis, facial pain, and cranial nerve III, IV, and VI palsies. Magnetic resonance imaging (MRI) and angiography revealed a spindle-shaped aneurysm in the cavernous segment of the right ICA with sphenoid sinus invasion. A balloon occlusion test demonstrated tolerance to ICA occlusion. Polymerase chain reaction analysis of sinus pus confirmed *Aspergillus fumigatus*. Despite antifungal therapy and sinus irrigation, the aneurysm enlarged. While flow diversion was being planned, the aneurysm ruptured, causing massive epistaxis and shock. Emergent PAO using a double catheter technique was performed, preserving collateral flow via the anterior and posterior communicating arteries. Postoperatively, the patient had no new neurological deficits, with only residual oculomotor palsy.

This case highlights the importance of early balloon occlusion testing in the management of fungal ICA aneurysms because of their high risk of rupture. Tight coil packing using a double catheter technique can minimize ischemic complications while preserving vital collateral circulation.

## Introduction

Infected cerebral aneurysms account for 0.7% to 6.5% of all cerebral aneurysms, the majority of which are bacterial in origin [1,2]. Fungal cerebral aneurysms are particularly rare, reflecting the overall rarity of fungal infections of the central nervous system, which account for only 0.5% of encephalitis cases [3]. There are two main etiologies of fungal cerebral aneurysms: hematogenous spread and direct invasion from sinusitis [4]. The latter is extremely rare and is associated with high mortality due to direct involvement of the cavernous segment of the internal carotid artery (ICA), although its natural history remains unclear [5,6]. While several reports have documented successful outcomes in unruptured cases treated with endovascular techniques or high-flow bypass [4,7], reports of survival following rupture are even more limited. In this report, we describe a case of a fungal ICA aneurysm caused by *Aspergillus* sinusitis that ruptured, resulting in massive epistaxis. The patient achieved a favorable outcome following successful emergent parent artery occlusion (PAO).

## Case presentation

An 80-year-old woman was referred to our hospital with complaints of right-sided ptosis and facial pain. She had begun chemotherapy for cervical cancer four months earlier. The following month, she developed drug-induced hepatitis and was treated with steroids for three months. She had no other significant medical history or medications.

At the time of hospitalization, she reported pain and a burning sensation on the right side of her face. Her body temperature was normal, and she was fully alert. The pupil of her right eye was dilated, the eye was fixed in the midline, and her vision was at the light perception level. These findings were suggestive of cranial nerve III, IV, and VI paralysis.

**Figure 1:**
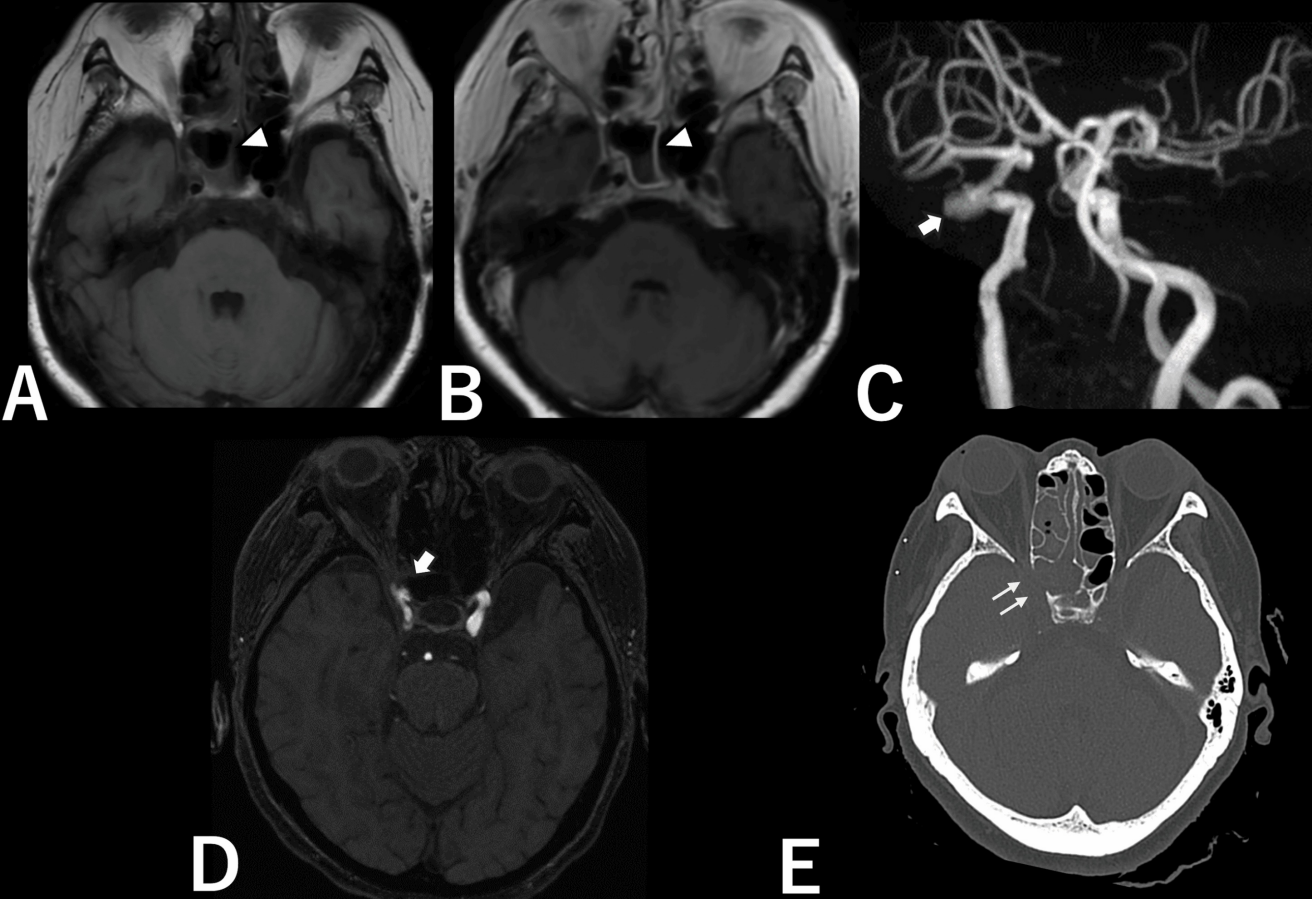
Imaging findings on admission. (A) T1-weighted MRI. (B) Gadolinium-enhanced T1-weighted MRI. White arrowheads in (A) and (B) indicate sinusitis. (C) MRA showing the aneurysm (white arrow). (D) MRA, axial view, showing the aneurysm (white arrow). (E) Head CT showing sphenoid bone loss in the aneurysm area (double white arrow).

A head MRI revealed a fluid collection with capsular contrast enhancement within the right sphenoid sinus (Figures 1A, 1B). Magnetic resonance angiography (MRA) showed a malformed wall aneurysm in the cavernous segment of the right ICA, protruding into the right sphenoid sinus (Figures 1C, 1D). Head CT demonstrated the absence of the sphenoid bone in the corresponding area (Figure 1E). Blood test results were negative for beta-D-glucan, and there were no notable abnormalities apart from a mildly elevated C-reactive protein level (2.73 mg/dL).

Fiberscope examination of the sphenoid sinus revealed a large amount of purulent discharge. Although cultures were negative, histopathological examination of the pus revealed filamentous fungi, and *Aspergillus fumigatus* DNA was later detected by polymerase chain reaction. The dura mater showed no signs of inflammation.

**Figure 2:**
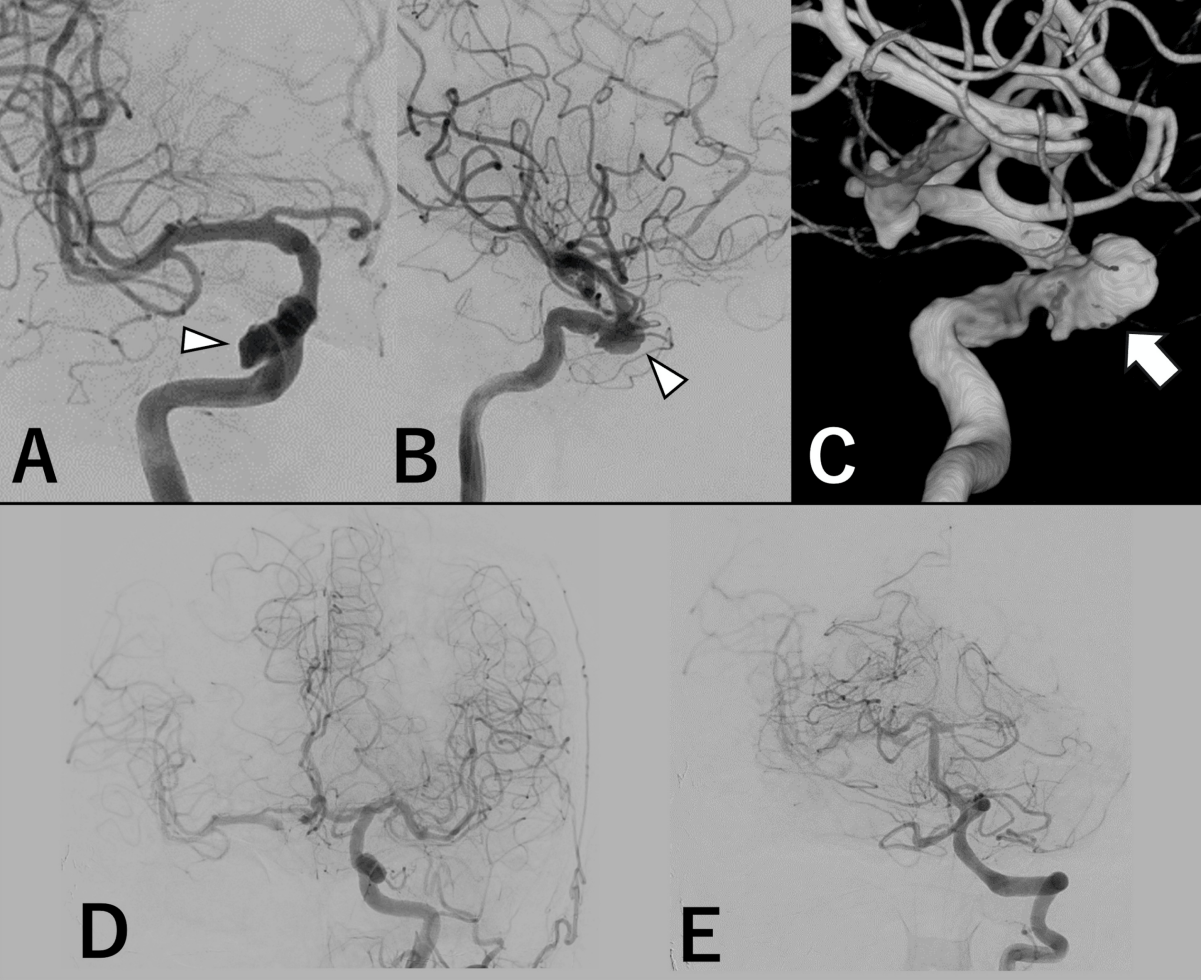
Angiographic findings. (A) Right internal carotid arteriography, anterior-posterior view. The white arrowhead shows the aneurysm. (B) Right internal carotid arteriography, lateral view. The white arrowhead shows the aneurysm.  (C) 3D-rotational angiography of the right internal carotid artery showing the aneurysm (white arrow). (D) Left internal carotid arteriography during balloon occlusion test. (E) Left vertebral arteriography after occlusion of the right internal carotid artery.

Right internal carotid angiography (ICAG) revealed a spindle-shaped aneurysm with a maximum diameter of 10 mm in the cavernous segment of the right ICA (Figures 2A-2C). During the angiography procedure, a balloon occlusion test (BOT) was performed. When the left ICA was occluded, the right cerebral hemisphere was immediately perfused via crossflow through the anterior communicating artery and the posterior communicating artery (PcomA) (Figures 2D, 2E). The PcomA joined the ICA slightly distal to the aneurysm. ICA occlusion did not cause any obvious neurological symptoms for 20 minutes, and the stump pressure ratio was 30%. Venous delay was less than one second. These results suggested that the patient had sufficient tolerance for right ICA occlusion. However, because of the low stump pressure, preserving antegrade blood flow was preferable. Additionally, considering the patient’s advanced age and immunocompromised status due to underlying malignancy, high-flow bypass surgery was deemed excessively invasive. Therefore, endovascular treatment was selected. Flow diverter (FD) stenting was planned as the first-line option, assuming the aneurysm’s configuration stabilized and the infection resolved. PAO with bypass surgery was also considered as a contingency in the event of rupture.

**Figure 3:**
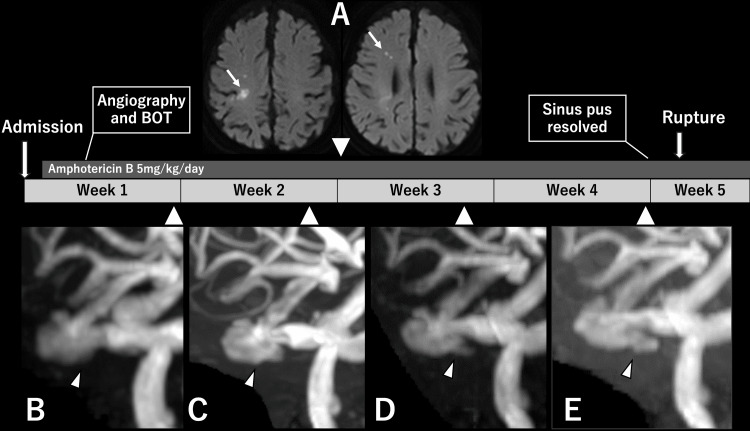
Timeline of MRI findings and clinical events after admission. (A) Diffusion-weighted image at the onset of left upper limb palsy, two weeks after admission, showing scattered cerebral infarcts (white arrow). (B) MRA at one week after admission. (C) MRA at two weeks. (D) MRA at three weeks. (E) MRA at four weeks. (B-E) The white arrowheads show the aneurysm. BOT: balloon occlusion test. MRI: Magnetic resonance imaging

The patient was treated with intravenous liposomal amphotericin B (5 mg/kg/day) along with weekly sinus irrigation and flushing. Two weeks after admission, she developed transient paralysis of the left upper limb. A head MRI revealed scattered cerebral infarcts in the right cerebral hemisphere (Figure 3A), suggesting embolization of fungal emboli from the infected focus in the right ICA. MRA also showed that the aneurysm appeared to be enlarging (Figures 3B-3E). By five weeks after admission, the patient was no longer discharging pus from the sinus, and normalization of C-reactive protein levels along with resolution of facial heat sensation indicated that the infection was well controlled. At this point, we were preparing to initiate antiplatelet therapy in preparation for FD placement. However, just a few days later, in the early morning, the patient suddenly developed massive epistaxis and went into shock, suggesting rupture of the aneurysm. She underwent tracheal intubation and required continuous blood transfusion. We decided to proceed with the emergent PAO.

Endovascular procedure

Under general anesthesia, an 8-Fr long sheath was inserted into the left femoral artery. An 8-Fr balloon guide catheter (Optimo EPD; Tokai Medical Products, Aichi, Japan) was advanced into the right cervical ICA, and the balloon was inflated. ICA occlusion resulted in a reduction of nasal bleeding. A distal access catheter (AXS Vecta 71; Stryker, Kalamazoo, MI, USA) was then positioned in the petrous segment of the right ICA.

**Figure 4:**
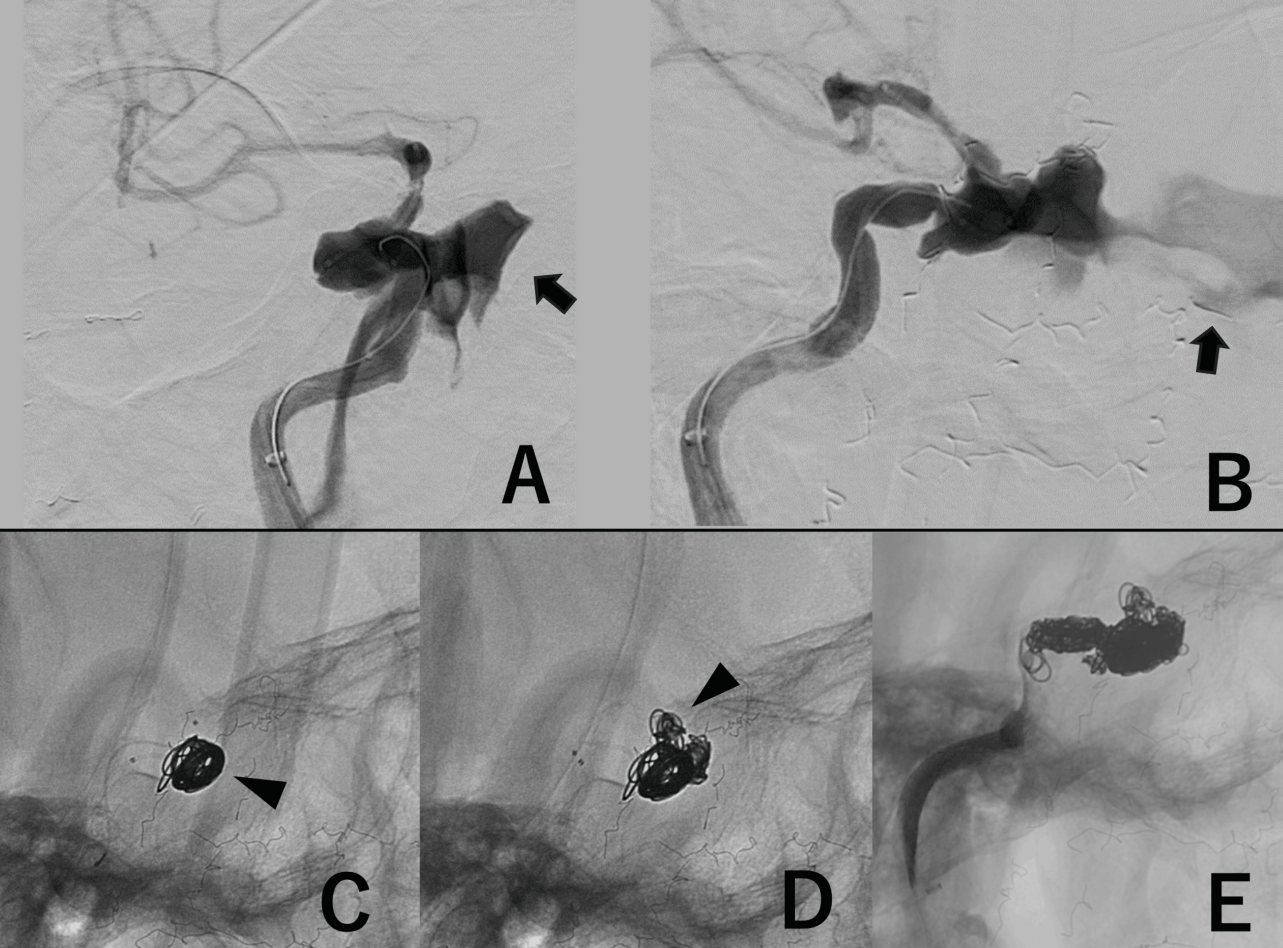
Angiography of endovascular treatment. (A) Right internal carotid artery, anterior-posterior view before embolization showing the extravasation (black arrow). (B) Right internal carotid artery, lateral view before embolization, showing the extravasation (black arrow). (C) Lateral view during embolization showing the first framing coil (black arrowhead). (D) Lateral view showing placement of the second coil immediately distal to the aneurysm (black arrowhead). (E) Final image after completion of parent artery occlusion.

**Figure 5:**
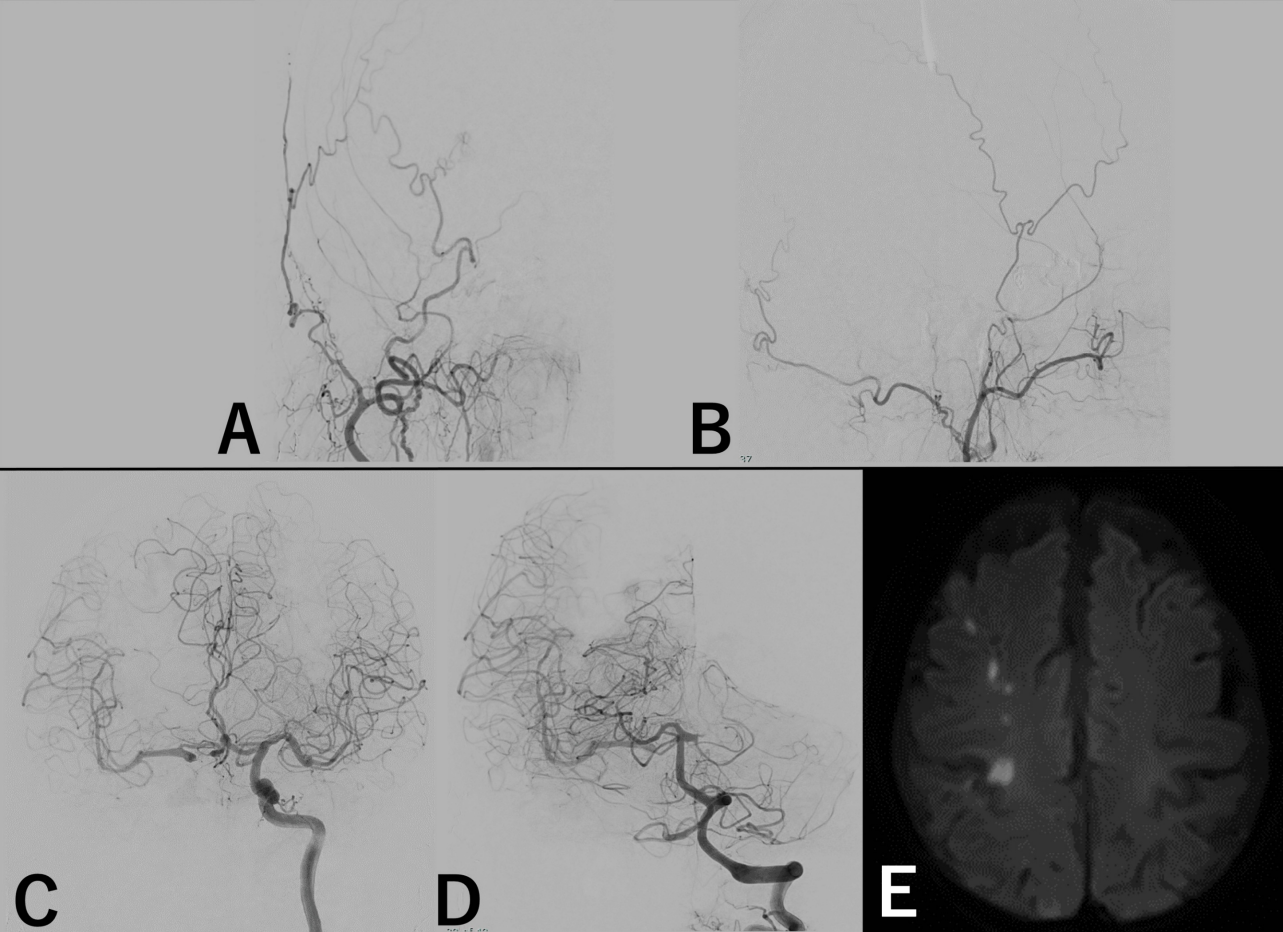
Images after embolization. (A) Right common carotid angiogram anterior-posterior view. (B) Right common carotid angiogram, lateral view. (C) Left internal carotid angiogram, anterior-posterior view. (D) Left vertebral artery angiogram, lateral view. (E) Postoperative head MRI (diffusion-weighted imaging).

Two microcatheters (Excelsior SL-10; Stryker) were inserted through the distal access catheter. One microcatheter was advanced distal to the aneurysm, and the other was positioned within the aneurysm using 0.014-inch micro guidewires (CHIKAI; Asahi Intecc, Aichi, Japan). ICAG demonstrated blood flow into the sinus cavity (Figures 4A, 4B). First, a framing coil was placed via the microcatheter within the aneurysm (Figure 4C). Then, the segment just distal to the aneurysm was packed using the second microcatheter, positioned in the ICA distal to the aneurysm, with the coil entangled with the first coil in the aneurysm (Figure 4D). Tight packing was performed within the aneurysm and on the proximal side using both microcatheters (Figure 4E). Subsequent right common carotid arteriography showed disappearance of antegrade flow in the right ICA (Figures 5A, 5B). The complete cessation of nasal bleeding indicated that hemostasis had been achieved. Follow-up left ICAG and vertebral arteriography confirmed cross-flow through the anterior communicating artery and PcomA to the peripheral territory of the right cerebral hemisphere (Figures 5C, 5D). No aneurysm was visualized retrogradely.

There were no new neurological deficits following the intervention. Postoperative MRI showed no increase in ischemic lesions suggestive of low perfusion (Figure 5E). The patient was transferred to the referring hospital for radiation therapy for endometrial cancer, with only oculomotor nerve palsy remaining. Her modified Rankin scale score remained at two, unchanged from admission.

## Discussion

We encountered a case of a ruptured fungal ICA aneurysm that achieved a favorable outcome with emergent PAO using the double catheter technique. It is rare for fungal sinusitis to invade the ICA within the cavernous sinus and form an aneurysm. Yamaguchi et al. reported that 18 such cases have been previously documented in the English literature [4]. Thirteen of these involved *Aspergillus* infections, as in our case, with the effect of *Aspergillus* elastase on the capillary wall cited as a contributing factor [4,8]. Other pathogens, such as *Candida albicans* and *Mucor* *species*, have also been reported as rare causes of fungal aneurysms [4]. These infections typically occur in individuals with underlying immunosuppressive conditions, such as diabetes mellitus, prolonged steroid use, or active malignancy [4].

The mortality rate of ruptured fungal ICA aneurysms is high. Among 18 reported cases, 13 ruptured, and only four patients survived [4,5,9-11]. Given the potential fragility and unpredictable progression of the aneurysmal wall due to fungal invasion, careful and frequent follow-up imaging is essential. Accordingly, the timing and indication for invasive treatment must be determined with great caution. According to the review by Yamaguchi et al. [4], all reported patients who received invasive treatment for unruptured aneurysms survived, highlighting the importance of early intervention to improve neurological outcomes. In this context, several reports have described successful treatment of unruptured aneurysms without neurological deterioration [4,7,12,13]. Yamaguchi et al. described a patient treated with high-flow bypass using the radial artery and ICA trapping, which allowed for early and reliable hemostasis [4]. However, concerns remain regarding the high invasiveness of the procedure, especially in immunocompromised and elderly patients. By contrast, several recent reports have documented successful treatment using FD stents [7,12,13]. This approach offers the advantage of being minimally invasive while preserving antegrade blood flow. Considering the patient’s general condition, we also selected FD placement as the first-line treatment. One of the main concerns with FD stent placement is the use of artificial material in infected vessels, which carries a risk of stent infection [9]. Adequate infection control is therefore essential. For example, Fujimi et al. administered antifungal therapy for approximately seven weeks prior to stent placement [7]. Another issue is the requirement for dual antiplatelet therapy before FD implantation. Because of the associated bleeding risks and potential delays in intervention, clinicians must carefully assess the risk of rupture when considering FD placement, as in our case.

There are few reports on the treatment of ruptured fungal ICA aneurysms [5,9-11]. In cases like ours, where rupture has occurred, we believe PAO should be considered because of the large volume of bleeding. Therefore, it is crucial to evaluate the patient’s tolerance to PAO by performing a BOT promptly after diagnosis to guide treatment planning. Regarding PAO for giant ICA aneurysms, Bechan et al. reported that when venous phase delay after BOT is less than one second, the positive predictive value for PAO tolerance is 98.9% (95% confidence interval: 93.2%-99.0%) [14]. In our case, the delay was less than one second, suggesting that the patient could tolerate PAO [14,15]. However, because the stump pressure was 30%, which was considered empirically insufficient to ensure adequate perfusion to the right cerebral hemisphere, we hesitated to perform preventive PAO for the unruptured aneurysm and planned FD treatment as the first-line option. Ultimately, a good outcome was achieved with PAO, and preventive PAO may have warranted earlier consideration.

In PAO, when the aneurysm is located in the cavernous segment of the ICA, it is essential to preserve the adjacent PcomA. We employed a double catheter technique with tight packing starting immediately distal to the aneurysm. The double catheter technique is particularly suitable for delicate coiling in cases where a critical branch arises from or near the aneurysm [16]. This approach allowed us to completely block blood flow to the aneurysm while preserving flow through the PcomA. As a result, the maintained flow through the PcomA helped prevent ischemic complications. Although the rupture created an urgent situation due to massive hemorrhage, careful packing using the double catheter technique proved highly effective.

## Conclusions

We experienced a case of a ruptured, fungal-infected ICA aneurysm. Given the high risk of rupture, it is important to assess PAO tolerance using the BOT at an early stage. One of the challenges of PAO in such cases is the proximity of the aneurysm to the PcomA, as the distance from an aneurysm located in the cavernous sinus to the PcomA is typically very short. Therefore, the double catheter technique, allowing for tight packing immediately distal to the aneurysm, was effective in preserving blood flow through the PcomA.
